# „Wut im Ohr“: Misophonie: Übersicht und aktueller Wissensstand

**DOI:** 10.1007/s00106-021-01072-7

**Published:** 2021-06-25

**Authors:** C. Schwemmle, C. Arens

**Affiliations:** Arbeitsbereich Phoniatrie, Pädaudiologie, klinische Audiologie, Universitätsklinik für Hals‑, Nasen- und Ohrenheilkunde, Otto-von-Guericke-Universität Magdeburg, Leipziger Straße 44, 39120 Magdeburg, Deutschland

**Keywords:** Geräuschempfindlichkeit, Emotionale Reaktion, Amygdala, Kognition, Triggerreduktion, Sound sensitivity, Emotional reaction, Amygdala, Cognition, Trigger reduction

## Abstract

Die Misophonie ist eine Intoleranz auf bestimmte Alltagsgeräusche. Hierbei fungieren als „Trigger“ „menschliche Körpergeräusche“, z. B. Schlucken/Schmatzen/Atemgeräusche oder Geräusche, die von Menschen, aber nicht vom menschlichen Körper erzeugt werden (z. B. Klicken Kugelschreiberknopf), ferner Tier‑/Maschinengeräusche. Die Betroffenen verspüren sofort eine negativ-emotionale Reaktion wie Wut, Aggression, Ekel u.a. Objektivierbare Veränderungen sind Herzfrequenzerhöhung und Blutdruckveränderungen. Die emotionale Reaktion ist individuell und hängt z. B. von Geräuschart, persönlicher Vorerfahrung, sozialem Kontext oder psychologischem Profil ab. Die Misophonie ist bisher als Krankheit nicht definiert und keinem offiziellen Diagnosesystem zugeordnet, sie scheint eine eigenständige Störung zu sein: Assoziationen bestehen u. a. mit Aufmerksamkeits‑/Zwangsstörungen, Tinnitus, Hyperakusis, Autismus-Spektrum-Krankheiten. Definitionskriterien wurden 2013 veröffentlicht; verschiedene, validierte Fragebögen wurden bisher zur Misophonieausprägung entwickelt. Studien mit funktionellen MRT-Untersuchungen des Kopfes zeigten eine übermäßige Aktivierung des anterioren Inselkortex (AIC) und seiner benachbarten Regionen, die für Emotionsverarbeitung/-regulation verantwortlich sind. Bisher gibt es keine randomisierten kontrollierten Studien zur Therapie. Einzelne Publikationen beschreiben kognitive Verhaltensinterventionen, Retrainingtherapien und Schallmaskierungssysteme. Zur Triggerreduktion werden Ohrstöpsel/Musikkopfhörer verwendet. Auch HNO-Ärzte können mit Misophoniepatienten konfrontiert werden, z. B. zur Klärung des Hörvermögens oder Beratung von Therapiemöglichkeiten. Der Bericht stellt eine Übersicht des aktuellen Wissensstands zur Misophonie sowie ihrer Diagnostik und Therapie dar.

## Abstract (en)

Misophonia is a devastating disorder. It is known as an affective sound-processing disorder characterized by the experience of strong negative emotions (e.g., anger, distress) in response to human sounds such as eating/swallowing/breathing. Other sounds produced by humans but not directly by human bodies can also be misophonic triggers (e.g. pen clicking) or environmental sounds (animal sounds/sounds of machines). The type of aversive triggers is individual. The reaction to trigger sounds can depend on many factors, such as assessment of the sound, personal experience, social context or psychological profile. However, there is currently no consensus in defining misophonia. Misophonia is also not yet classified by any official diagnostic system, although it seems to be a separate disorder. There are also associations with other disorders such as activity disorders, tinnitus, hyperacusis, and autism spectrum disorders. In 2013, the first definition criteria were published for the diagnosis of misophonia. Specifically, fMRI showed abnormal activation of the anterior insular cortex (AIC) and other brain areas responsible for the processing and regulation of emotions. To date, no randomized controlled trials evaluating treatments have been published. The use of cognitive and behavioral interventions have been reported as well as external sound systems and sound masking systems as known in the tinnitus retraining therapy. Sufferers try to minimize the trigger sounds by wearing ear plugs or music headphones. Otolaryngologists may also encounter patients with symptoms of misophonia, e.g., when hearing screening is requested or advice should be given on different therapeutic options. This report provides an overview of the current state of knowledge in misophonia and its diagnosis and treatment.

Heinz Funke war fünf, als er die Schuhe seiner Mutter mit einer Schere zerstörte. Er ritzte die Schnallen an und behauptete, der Hund habe sie zerkaut. Heute ist Heinz Funke 53. Er ist Fachkraft für Lagerlogistik, ist aber arbeitsunfähig. Er sieht einen Zusammenhang zwischen seiner jetzigen Verfassung und der Sache mit den Schuhen: Funke konnte simple Alltagsgeräusche bereits in der Kindheit nicht ertragen, z. B. die klackernden Schuhabsätze seiner Mutter; außerdem explizit Kaugeräusche, sodass z. B. das Essen in Anwesenheit anderer nicht möglich war (und bis heute nicht ist). Heinz Funke leidet unter Misophonie, was er aber erst seit einigen Jahren weiß. Vorher dachte er immer, er sei „nicht richtig im Kopf“ [28].

## Geräuschüberempfindlichkeit

Prinzipiell lassen bestimmte Geräusche jeden zusammenzucken; man denke z. B. an das schrille Kratzen der Kreide an der Tafel oder die penetrant quietschenden Bremsen des einfahrenden Zuges in den Bahnhof. Geräusche, die durch ihre Intensität förmlich zu Schmerzen, Gänsehaut und zusammengezogener Nackenmuskulatur führen können. Doch wann ist diese Wahrnehmung pathologisch?

Unter dem Begriff der Geräusch(über)empfindlichkeit können nosologisch differente Phänomene subsumiert werden, die alle dadurch gekennzeichnet sind, dass die Betroffenen auf normale Umgebungsgeräusche mit *hohem* subjektivem Leiden und oft mit Vermeidungsverhalten reagieren. International wird die Geräuschüberempfindlichkeit als „hyperacusis“ oder „decreased sound tolerance“ im Sinne eines Sammelbegriffs verwendet [25]. Schaaf et al. geben eine gute Übersicht zu diesen Geräuschüberempfindlichkeiten [53]. Hierzu gehören:

1. die Hyperakusis
2. die Phonophobie
3. die Misophonie

### Hyperakusis

Der Begriff „hyper“ (griechisch, über), akusis („akuo“: ich höre) beschreibt eine negativ bewertete, subjektive Überempfindlichkeit gegenüber Geräuschen *normaler* Lautstärke (unterhalb 70–80 dB HL) über das *gesamte *Frequenzspektrum menschlicher Hörwahrnehmung und führt unmittelbar zu einer psychischen Reaktion wie motorische Unruhe, Konzentrationsschwankungen, Angst, Panik und Frustration. Körperliche Symptome sind physiologische Schreckreaktionen wie Zu- oder Abnahme des Blutdrucks, Brady- oder Tachykardie, verstärkte Schweißsekretion, Mundtrockenheit, im Ohrbereich lokalisierte Schmerzempfindung, Zunahme des Muskeltonus insbesondere im Schulter-Nacken-Bereich und Abwendung von der Geräuschquelle (Kopf, Körper). Ein eventuell vorbestehender Tinnitus kann für mehrere Stunden bis Tage verstärkt sein.

Die Hyperakusis ist eine Abneigung gegen „alle“ lauten Geräusche, die von Nichtbetroffenen als unbedeutend wahrgenommen werden (z. B. Staubsauger, Telefonklingeln, Waschmaschinenschleudergang). Sämtliche Geräusche des Alltags, können beim Betroffenen die genannten Symptome auslösen [53].

### Phonophobie

Phonophobie (griechisch „phōnē“: Sound, Stimme, und „phobos“: Scheu, Furcht, Angst, zu altgriech. „phobeisthai“: fürchten, scheuen).

Bei der Phonophobie handelt es sich – anders als bei der Misophonie – nicht um eine Abneigung definierter Geräusche, sondern um eine *allgemeine *Angst/Abneigung vor Geräuschen [52].

Phonophobie-Betroffene haben meistens konstant Angst vor möglichen lauten Geräuschen und auch bereits *vor* dem möglichen Eintreten eines akustischen Phänomens. Der Knall oder die laute Musik müssen also aktuell gar nicht vorhanden sein, um Angst auszulösen.

### Misophonie

Die Misophonie (griechisch „miso“: Hass oder Abneigung, „phōnē“: Sound, Stimme, englisch „hatred of sounds“), der dieser Beitrag gewidmet ist und im nachfolgenden Kapitel weiter erörtert wird, ist eine selektive Geräuschempfindlichkeit. Unmittelbar nach Wahrnehmung von sog. Triggergeräuschen reagieren die Betroffenen reflexartig z. B. mit Wut, Irritation, Aggression u. a. Es findet sich keine eindeutige physikalische Eigenschaft des Stimulus’ [33]. Stattdessen scheinen die Bedeutung, der soziale Kontext oder die Interpretation des Auslösers die Reaktion auf diese Geräusche zu beeinflussen [6, 54, 63]. Die aversiv erlebten Geräusche werden bereits bei geringer Lautheit als überlaut, störend und bedrohlich empfunden, andere Geräusche mit vergleichbarem Frequenzspektrum verursachen keine Symptome. [33, 53, 65].

## Misophonietrigger

**Figure 1:**
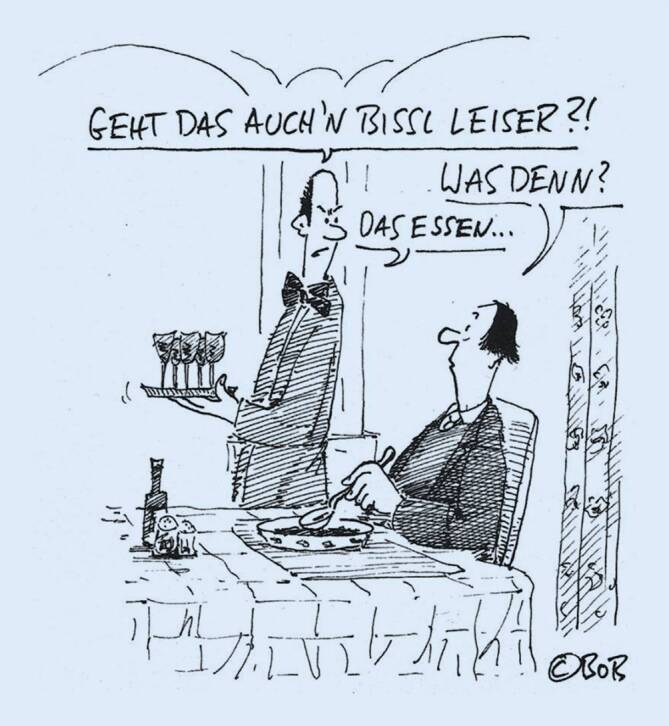

Die Auslöser („Trigger“) einer misophonen Reaktion sind meistens „menschliche Körpergeräusche“, vorrangig Essgeräusche wie schlucken/schmatzen/schlürfen/kauen sowie Atemgeräusche/schniefen ([5, 29, 31, 46]; Abb. 1).

Auch Alltagsgeräusche können Triggergeräusche sein, die von Menschen, aber nicht direkt vom menschlichen Körper erzeugt werden, wie z. B. Klicken mit dem Kugelschreiberknopf, mit Papier rascheln, Geräusche von Schuhabsätzen, außerdem Geräusche, die von (Haus‑)Tieren [12, 46] produziert werden, sowie Maschinengeräusche u. Ä. (z. B. Toilettenspülung) [46]. Eine Triggerauslösung kann auch bestehen, wenn diese Trigger z. B. im Fernsehen wahrgenommen werden [19].

Neben akustischen Triggern können – seltener – auch visuelle Trigger Auslöser sein wie z. B. das Schlenkern des übergeschlagenen Beins beim Sitzen, der Anblick der Lippenbewegung beim Essen [19, 35, 54], auch wenn das Essensgeräusch durch z. B. größere Entfernung nicht gehört werden kann [11, 29, 65]. Die Symptomatik fängt meist mit einem bestimmten Triggergeräusch an und kann sich längerfristig auf weitere Geräusche ausweiten, nach Rouw et al. bei über 70 % der Betroffenen [37, 50].

Nachfolgende Tab. 1, modifiziert nach Jager [29], zeigt die wichtigsten Triggerauslöser und ihre prozentuale Verteilung.

**Table 1:** 

Triggerausprägung in %	Graduierung Ärger/Wut (Skala 0–4)
*Essensgeräusche (96* *%)*	
Schmatzen	3,3
Chips o. Ä. essen	2,9
Kaugummi kauen	2,8
Schlürfen	2,7
In einen Apfel beißen	2,5
Trinkschluckgeräusche	2,4
*Atem‑/Schniefgeräusche (85* *%)*	
Schnarchgeräusche	2,6
Schniefgeräusche	2,5
Atemgeräusche	2,3
Niesen	0,9
*Körperbewegungen (78* *%)*	
Schaukeln von Beinen	2,0
Geige spielende Finger	1,5
Kratzbewegung Finger	0,7
*Geräusche durch Finger (74* *%)*	
Fingernägel bearbeiten	2,0
Kugelschreiber klicken	1,9
Finger auf Oberfläche tippen	1,6
Schreiben auf Tastatur	1,7
Essbesteck verwenden	1,5
*Mund/Kehlgeräusche (69* *%)*	
Räuspern	1,7
Husten	1,6
Flüstern	1,4
Küssen	1,3
Gähnen	1,1
*Umgebungsgeräusche (59* *%)*	
Musik von Nachbarn	2,0
Sich unterhaltende Nachbarn	1,6
Maschinengeräusche (Uhr usw.)	1,2
Telefonklingeln	1,1
Tiergeräusche	1,0
*Raschelgeräusche (42* *%)*	
Plastiktütenrascheln	1,3
Seiten umblättern	0,6

## Misophoniereaktion

Die Misophoniereaktion ist individuell unterschiedlich. Sie steht meistens in einem grotesken Missverhältnis zu dem wahrgenommenen Geräusch, auch weil die Geräuschintensität eine deutlich untergeordnete Rolle spielt und es sich um „von klein auf“ gewohnte Geräusche handelt, die man ja auch selbst produziert (wie z. B. Kaugeräusche).

Die Misophoniker verspüren bei Wahrnehmung „ihrer“ Triggergeräusche objektivierbare Veränderungen wie Herzfrequenzerhöhung, Schweißausbrüche, Blutdruckerhöhungen und Atemnot [19]. Die emotionale Reaktion zeigt sich einschießend häufig durch Aversion, Irritation, Aggression (auch Wut), überstürztes Weglaufen (z. B. vom Esstisch), Ekel und andere, ausschließlich negativ-emotionale Assoziationen [5, 12, 13, 19, 29, 41, 54, 65]. Auch Schlagen des „Verursachers“ (11,9 %), verbale Aggression (28,6 %) oder Attacken gegen Gegenstände in der Umgebung (16,7 %) wurden beschrieben [54]. Emotional-gedankliche Reaktionen auf den Triggerverursacher beschreiben u. a. „Ich hasse diese Person“, „warum tut er/sie mir dies an“, „die Person sollte aufmerksamer sein, was sie anderen antut“ [19]. Soziale Konflikte wie z. B. in der Konstellation Eltern – Kind oder in einer Partnerschaft sind vorprogrammiert: Triggerinduzierte Reaktionen sind häufig ausgeprägter, wenn der Trigger von Angehörigen und engen Vertrauten „produziert“ wird [19]. Trotzdem erkennen Misophoniker, dass ihre Reaktion unbegründet und der Verlust der Selbstkontrolle inakzeptabel war [54]. Teilweise werden Bewältigungsstrategien entwickelt bzw. Veränderungen im Alltag von Misophonikern vorgenommen, die den Umgang mit den Triggerreaktionen erleichtern [67].

In vielen Fällen vermeiden Misophoniker Situationen, die das Auftreten des misophonen Reizes wahrscheinlich machen (z. B. nicht zusammen essen). 7 % der Misophoniker vermeiden es außerdem, den/die Trigger selbst zu produzieren [19].

Das Ausmaß der Triggerreaktion ist auch abhängig von der Möglichkeit, sich zurückzuziehen (öffentlicher Platz versus Autoinsasse) [19]. Betroffene zeigen oft ein Vermeidungsverhalten mit Einschränkung des Bewegungsradius, brechen soziale Kontakte ab [13, 19], verlassen im Extremfall kaum noch den eigenen Wohnbereich, fahren nicht mit öffentlichen Verkehrsmitteln, brechen Ausbildungen oder das Studium ab und sind nicht oder nur sehr begrenzt arbeitsfähig [28, 53], Kinder mit Misophonie können nicht mehr in der Lage sein, mit der Familie zu essen [19, 28] oder zur Schule zu gehen [9]. Vereinzelt bestehen suizidale Gedanken [19]. Ist die Triggervermeidung nicht möglich, oder ein Triggergeräusch kommt unerwartet, ergibt sich entweder „Flucht oder Konfrontation“. Der Begriff „fight-or-flight response“ (deutsch: „Kampf-oder-Flucht-Reaktion“) umschreibt dieses Phänomen. Der US-amerikanische Physiologe Walter Cannon (1871–1945) prägte diesen Begriff, der neurobiologische Abläufe bei Tieren auf Bedrohung beschreibt. Ausgangsbasis waren Auswirkungen von posttraumatischen Belastungsstörungen bei Soldaten im/nach dem Ersten Weltkrieg [8]. Die Cannon-Bard-Theorie, die er mit seinem Schüler Philip Bard entwickelte, beschreibt, dass ein Emotionsreiz zwei gleichzeitig ablaufende Reaktionen induziert: die physiologisch induzierte und die Emotionswahrnehmung. Keine der beiden Reaktionen bedingt die andere.

Substanzen wie Alkohol führen möglicherweise zu einer Verminderung der Triggerreaktion, Koffein vermutlich zu einer erhöhten Reaktion [50].

Schaut man in die internationale Literatur, zeigt sich eine Imbalance zwischen popularmedialen Interneteinträgen und der wissenschaftlichen Literatur, hier exemplarisch bei „PubMed“ mit dem Stichwort „misophonia“ 65 Einträge, unter den Stichworten „misophonia“ und „german“ 0 Einträge (Zugriff 16.04.2020), wenn auch kurz nach dem Zugriff 2020 neue Arbeiten veröffentlicht wurden [12, 13, 29, 62]. In den gängigen Suchmaschinen wie „Google“ stößt man bei dem Stichwort „misophonia“ auf 554.000 Einträge, unter dem Stichwort „Misophonie“ auf 45.100 Einträge (Zugriff 16.04.2020). Vor allem Misophonie-Foren sind möglicherweise deshalb so präsent, weil der Austausch von Betroffenen immer dann auch eine große Rolle spielt, wenn die wissenschaftliche und medizinische Auseinandersetzung und Therapie zu diesem Thema eher rar ist.

Wissenschaftlich zeigte sich seit 2013 ein größeres Interesse [50]. Überwiegend wurden Einzelfallbeschreibungen oder kleinere Fallstudien publiziert [4, 6, 14, 22, 35, 37, 47, 66, 5], eine umfassende Übersicht gab Taylor 2017 [65]. Jager veröffentlichte die bisher größte Studie mit 575 Teilnehmern [29].

## Historie

In den 1990er-Jahren beschrieb die Audiologin Marsha Johnson erstmals das „selective sound sensitivity syndrome“ [63, 12, 36] oder „soft sound sensitivity“ [4].

Die Neurowissenschaftler Pavel und Margret Jastreboff verwendeten Anfang der 2000er-Jahre für den „Hass auf Geräusche“ („hatred of sounds“) den Begriff „Misophonie“ [31]. Bereits in den 1990er-Jahren, entwickelten sie ein audiologisch-neurologisches Modell der Tinnitusentstehung als komplexes Geschehen einer Phantomperzeption, das auch für die Misophoniewahrnehmung konzeptionell beschrieben wurde [30, 46]. Eine Koexistenz der Misophonie und der Hyperakusis als allgemeine Geräuschüberempfindlichkeit wurde frühzeitig postuliert [21, 26, 33, 34]. Schröder et al. prägten 2013 erstmals Definitionskriterien aufgrund einer eigenen Studie [54], die von Dozier 2017 erweitert wurden [17, 63]. Diese Kriterien sind nicht unbestritten, weil u. a. das Kriterium „Zorn“ nicht immer zuträfe und dies in der Diagnose der Misophonie berücksichtigt werden müsste [41]. Sowohl der Begriff „selektive Geräuschempfindlichkeit“ als auch „Misophonie“ sind in der Literatur präsent [63], Letzterer ist jedoch gebräuchlicher geworden [61].

Die Misophonie ist bisher als Krankheit nicht definiert und keinem offiziellen Diagnosesystem zugeordnet, weder dem diagnostischen und statistischen Handbuch für psychische Störungen, 5. Auflage (DSM-V) noch der Internationalen Klassifikation von Krankheiten, 11. Auflage (ICD-11) [1, 12, 29].

## Häufigkeit, Symptombeginn, Geschlechtsverteilung

Über die Inzidenz und Prävalenz kann bisher nur spekuliert werden [56]. Einige Forscher vermuten ein „relativ häufiges“ Auftreten [10, 65]. Im Hinblick auf die Koexistenz verschiedener Hörpathologien bei Misophonie beziehen sich die Schätzungen häufig auf das Konstrukt Geräuschüberempfindlichkeit und/oder Tinnitus. Fast 20 % von 483 amerikanischen Psychologiestudierenden [67] und 6 % von 415 chinesischen College-Studierenden [68] gaben an, unter klinisch signifikanten misophonen Symptomen zu leiden, andere Schätzungen postulierten 3 % in der Allgemeinbevölkerung [42].

Misophone Reaktionen treten oft erstmals in der späten Grundschulzeit [19] oder frühen Pubertät auf [67]. Verschiedene Studien gaben im Mittel 12 Jahre an [20, 39], aber auch über die Symptomatik bei einem Fünfjährigen wurde berichtet [28, 39] sowie in Interviews „so lange, wie ich schon denken kann“ [19]. Schröder beschrieb 2013 eine große Bandbreite der Erstmanifestation von 2–38 Jahren [54]. Neuere Veröffentlichungen postulieren die Erstmanifestation um das 14. Lebensjahr [29]. Möglicherweise ist die Gefahr für eine Misophonie einerseits durch sensible/vulnerable Phasen der Gehirnentwicklung, andererseits durch bereits bestehende Erfahrung/Bewertung, Lernen und Prägung zu Anfang der hormonellen Veränderung in der Pubertät am höchsten.

Auffällig ist der deutlich spätere Diagnosezeitpunkt (überwiegend im Erwachsenenalter), der von Schröder mit 19–62 Jahren beziffert wurde [54]. Die Symptomatik fängt meist mit einem bestimmten Triggergeräusch an und kann sich längerfristig auf weitere Geräusche ausweiten [50, 58].

Frauen sind vermutlich häufiger betroffen. Cavanna berichtete zwar von einem balancierten Verhältnis von Frauen zu Männern (55/45 %) [10, 65], aber andere von einem 2:1-Verhältnis [13, 19] bis 3:1 [29, 56]. Wu et al. beschrieben einen Anteil von über 83 % von Frauen [67].

## Ursachen

Die Misophonie wird im Kontext audiologischer, psychiatrischer, kognitiv-verhaltensspezifischer und neurologischer Ursachen eingeschätzt. Inwiefern sie als eigenständige Krankheit oder als Kosymptom anderer Krankheiten zu bewerten ist, ist bisher ungeklärt [5].

Genetische Faktoren als additive Ursache könnten prinzipiell möglich sein. Eine Analyse mit 15 Familienmitgliedern und Misophonie beschrieb eine mögliche autosomal-dominante Vererbung [51]. Danesh und Aazh berichteten über die Häufung von Chromosom-5q34-Veränderungen bei Misophoniepatienten, die eine genetische Analyse mit kommerziell erhältlichen Chromosomen-Kits durchführen ließen [12].

Verschiedene Studien beschrieben Komorbiditäten zu Tinnitus/Hyperakusis [31], Migräne [64], Störungen aus dem autistischen Formenkreis [3, 24], posttraumatischen Störungen [2], Borderline-Störungen [49], bipolaren Störungen/Schizophrenien [7, 22], Angst- und Zwangsstörungen [22], Essstörungen [37], Depressionen und Aufmerksamkeitsdefizitsyndromen [23, 29]. Einzelfallkasuistiken berichteten von einem Kind mit Tourette-Syndrom [66], Zwangsstörung und Misophonie [27]. Zhou et al. befragten 415 chinesische College-Studenten: Assoziationen bestanden mit sensorischer Empfindlichkeit, Zwangsstörungen, Angstzuständen und depressiven Symptomen [68]. Taylor postulierte die Misophonie zusammenfassend als psychiatrisches Syndrom [65], das von jüngeren Veröffentlichungen gestützt wird [29] und vermutlich mit einer verminderten kognitiven Kontrolle assoziiert ist [13]. Es gilt aber als wahrscheinlich, dass das Störungsbild nicht auf eine einzige Ursache zurückzuführen ist.

## Neuronale Korrelate

Nach Jastreboff [33] gibt es Symptomgemeinsamkeiten von Misophonie und Tinnitus durch eine abnormale neuronale Aktivität mit nachfolgend negativ-assoziierter Bewertung des Organismus. In subkortikalen Prozessen ist die Abschwächung oder Verstärkung eines Reizes davon abhängig, ob das betreffende Signal zu diesem Zeitpunkt von Bedeutung ist. So ergeben sich prägende neuronale Lernprozesse und Interaktionen, die auch von Stimmungslage, persönlicher Betroffenheit u. a. abhängig sind. Ein bisher irrelevantes akustisches Signal kann jederzeit plötzlich die Bedeutung eines wichtigen Signals bekommen und als Mustererkennung über das limbische System eine emotionale Bewertung erhalten. Edelstein postulierte, dass ein zugrunde liegender Mechanismus für eine neurobiologische Reaktion Ähnlichkeiten mit synästhetischen Wahrnehmungen hat, eine sensorische Wahrnehmung („inducer“) die Aktivierung verschiedener neurologischer Systeme gleichzeitig konkurrierend initiiert [19, 5]. Kritisch ist anzumerken, dass eine Synästhesiewahrnehmung überwiegend stabil bleibt, was klinisch für die Misophonie oftmals nicht zutrifft [5].

Eine EEG-Studie 2014 zeigte eine geringere N1-Aktivität hinsichtlich ereigniskorrelierter Potenziale bei Misophoniebetroffenen, das möglicherweise auf ein neurobiologisches Defizit in der auditiven Verarbeitung hinweist. Unklar blieb, ob dieses Defizit allgemein bei psychiatrischen Patienten auftritt oder nur speziell bei Misophoniebetroffenen, außerdem, ob das Defizit auch auf Dysfunktion der kortikalen Kontrolle zurückzuführen ist [55]. Kumar et al. postulierten 2017 aufgrund von funktionellen Magnetresonanztomografie-Daten (fMRT) eine erhöhte neuronale Vernetzung im ventromedialen Präfrontalkortex und die Aktivierung des anterioren insulären Kortex. Der Präfrontalkortex ist an der Verarbeitung sensorischer Informationen beteiligt und beeinflusst die Funktion anderer Gehirnbereiche (z. B. Areale Gedächtnisverarbeitung und -speicherung; Amygdala für emotionale Konditionierung, Verarbeitung für Kampf-Flucht-Reaktion sensorischer Stimuli). Der insuläre Kortex ist vor allem an der Bewertung/Verarbeitung sensorischer Informationen und Emotionen beteiligt [40]. Andere fMRT-Studien fanden Korrelationen zwischen Misophonie und perfektionistischem bzw. zwanghaftem Verhalten [20].

## Diagnostik

Die Diagnose ist eine klinische Diagnose, die aufgrund der Anamnese und der Erhebung der Symptomatik gestellt wird. Hilfsmittel sind Checklisten, Fragebogenverfahren und Interviews. HNO-ärztlich sind begleitend HNO-Untersuchung, objektive und subjektive audiologische Diagnostik zu möglichen Kofaktoren der Hörpathologie wie periphere Höreinschränkungen, Tinnitus, auditorische Neuropathien sowie Beurteilung der Unbehaglichkeitsschwelle und Lautheitsskalierung, psychoakustische Tests unter Störschallbedingungen sinnvoll. Überschwellige Tests, Stapediusreflexdiagnostik sind bezüglich ihrer tonalen Lautstärke nicht ratsam.

Schröder et al. definierten 2013 [54] die Misophonie anhand von 6 Punkten, die von Dozier 2017 modifiziert wurden. Beide Kriterien-Sets basieren auf Forschungsergebnissen und Fallstudien von Menschen mit Misophonie, wurden bisher aber nur begrenzt empirisch verifiziert [63]. Nachfolgend zeigt Tab. 2 die Kriterien von Schröder, modifiziert von Dozier [17]:

**Table 2:** 

A)	Das Vorhandensein oder die Antizipation einer bestimmten sensorischen Erfahrung wie z. B. eines Geräusches, eines Anblicks oder eines anderen Reizes (z. B. Essgeräusche, Atemgeräusche, Maschinengeräusche, Beinbewegung, Vibration) provoziert eine impulsive, aversive physische und emotionale Reaktion, die typischerweise mit Irritation oder Ekel beginnt und schnell in Wut übergeht
B)	Der Reiz löst eine sofortige physische Reflexantwort aus (Aktivität der Skelett- oder viszeralen Muskulatur, sexuelle Reaktion, Wärme, Schmerz oder andere körperliche Empfindungen). Beachte, dass die physische Reaktion nicht immer identifiziert werden kann, aber die Präsenz einer sofortigen physischen Reaktion kann genutzt werden, um die Symptome klarer als Misophonie zu identifizieren
C)	Eine moderate Reizdauer (z. B. 15 s) löst allgemeine physiologische Erregung aus (z. B. Schwitzen, erhöhte Herzfrequenz, Muskelanspannung)
D)	Fehlregulation von Gedanken und Emotionen mit seltenen, aber potenziell aggressiven Ausbrüchen. Bei Kindern können aggressive Ausbrüche häufig sein
E)	Die negative emotionale Erfahrung wird später als übertrieben, unvernünftig, oder unverhältnismäßig zu den Umständen oder dem provozierenden Stressor anerkannt
F)	Die Person neigt dazu, die misophone Situation zu vermeiden, oder wenn nicht, erträgt sie die misophonische Reizsituation mit Unbehagen oder Leid
G)	Emotionales und physisches Erleben, Vermeidung und Bemühen zu vermeiden führen zu erheblichem Leid oder bedeutsamen Beeinträchtigungen im Alltag des Betroffenen. Zum Beispiel ist es schwierig für die Person, bei der Arbeit Aufgaben auszuführen, am Unterricht teilzunehmen, an Routinetätigkeiten zu partizipieren oder mit bestimmten Personen zu interagieren

Fragebogeninventare geben hilfreiche Aussagen bezüglich Vorhandensein/Ausprägung. Alle beschriebenen sind in englischer Sprache und wurden von Seebeck in das Deutsche übersetzt [58]. Eine Validierung auch in der englischen Fassung besteht nicht.

1. *Misophonie-Selbstbewertungsfragebogen (Misophonia Assessment Questionnaire, MAQ):* Von M. Johnson entwickelt, modifiziert von Dozier [18, 62], 21 Fragen, Punktescore 0–3/Frage, Summenscore zur Beurteilung nicht/kaum bis sehr deutlich ausgeprägt [45].
2. *Misophonie-Fragebogen (Misophonia Questionnaire, MQ*): Im englischsprachigen Sprachraum verbreitet, Kombination aus Symptombeurteilung/Emotion/Verhalten, 17 Items [67].
3. *Misophonie Aktivierungsskala (Misophonia Activation* *Scale, MAS‑1*): Von Fitzmaurice auf der Internetplattform misophonia-uk.org publiziert [s. auch in 62], Stufe 0–10, Einordnung der eigenen Reaktionsschwere auf Trigger.
4. *Amsterdam Misophonieskala (Amsterdam-Misophonia-Scale, A‑MISO-S)* [54], Adaption Yale-Brown Obsessive Compulsive Scale (Y-BOCS) für Zwangsstörungen. Beurteilung der Misophoniebeeinflussung zeitlich, Beeinflussung von Lebensqualität und Arbeitsleistung. Häufig verwendet, ökonomisch rasch durchführbar. Nachfolgend exemplarisch dargestellt in Tab. 3.

**Table 3:** 

**Frage 1: Wieviel Ihrer Zeit wird durch die Auswirkung Ihrer misophonischen Trigger beansprucht? Wie oft kommen die Trigger selbst oder Gedanken über die Trigger vor?**
0: Vernachlässigbar
1: Gering. Weniger als 1 h pro Tag. Seltene Trigger (oder Gedanken darüber), d. h. weniger als 5 Mal täglich
2: Mäßig. 1–3 h täglich. Häufige Trigger (oder Gedanken darüber), d. h. mehr als 8 Mal täglich
3: Akut. Zwischen 3 und 8 h täglich. Sehr häufige Trigger (oder Gedanken darüber)
4: Extrem. Mehr als 8 h täglich bzw. nahezu konstante Trigger (oder Gedanken darüber)
**Frage 2: Wie wirken sich Ihre misophonischen Trigger auf Ihr Sozial- und Berufsleben aus? (Gibt es Aktivitäten, die Sie aufgrund der Trigger vermeiden? Wenn Sie zur Zeit nicht arbeiten, bewerten Sie, wie sich die Trigger auf Ihr Arbeitsleben auswirken würden.)**
0: Vernachlässigbar
1: Gering. Geringfügige Auswirkung auf das Sozial‑/Berufs‑/Schulleben. Gesamtleistung ist nicht beeinträchtigt
2: Mäßig. Gewisse Beeinträchtigung auf das Sozial- und Berufsleben, aber immer noch beherrschbar
3: Akut. Wesentliche Beeinträchtigung auf das Sozial- und Berufsleben
4: Extrem. Arbeitsunfähig
**Frage 3: Bewerten Sie das Stressniveau, das Ihre misophonischen Trigger verursachen. (Ziehen Sie nur die Emotionen wie Irritation, Wut oder Ekel in Betracht, die direkt mit Ihren misophonischen Triggern verbunden sind, nicht generelle Irritation, die auf andere Zustände zurückzuführen ist.)**
0: Vernachlässigbar
1: Gering. Gelegentlicher Stress/Irritation
2: Mäßig. Störende, aber beherrschbare Irritation/Wut/Ekel
3: Akut. Sehr störende Irritation/Wut/Ekel
4: Extrem. Nahezu konstante und störende Irritation/Wut/Ekel
**Frage 4: Wie sehr bemühen Sie sich, Ihre Trigger auszuhalten? (Wie oft versuchen Sie, die Trigger zu ignorieren? Bewerten Sie hier nur Ihr Bemühen, die Trigger auszuhalten, und nicht, ob Sie erfolgreich Ihre Gedanken kontrollieren.)**
0: Ich bemühe mich immer, meine Trigger auszuhalten. Meine Symptome sind minimal, und es bedeutet keine Mühe für mich
1: Ich bemühe mich meistens, meine Trigger auszuhalten
2: Ich bemühe mich bis zu einem gewissen Grad, meine Trigger auszuhalten
3: Ich gebe widerwillig nach und versuche nicht, meine Trigger auszuhalten
4: Ich gebe allen meinen Impulsen widerstandslos nach
**Frage 5: Wie sehr können Sie Ihre Gedanken über Ihre misophonischen Trigger kontrollieren? (Wie erfolgreich sind Sie dabei, Ihr Denken umzusteuern?)**
0: Komplette Kontrolle
1: Viel Kontrolle. Ich kann normalerweise damit aufhören, an meine misophonischen Trigger zu denken
2: Mäßige Kontrolle. Ich kann manchmal damit aufhören, an meine misophonischen Trigger zu denken
3: Wenig Kontrolle. Ich schaffe es meistens nicht, damit aufzuhören, an meine misophonischen Trigger zu denken, und kann meine Aufmerksamkeit nur schwer auf etwas anderes umlenken
4: Keine Kontrolle. Meine Gedanken über die misophonischen Trigger sind völlig unfreiwillig, und ich kann sie nur sehr selten abschalten
**Frage 6: Vermeiden Sie bestimmte Aktivitäten, Orte oder Leute aufgrund Ihrer Misophonie? (Zu welchem Grad gebrauchen Sie laute Geräusche, z. B. Musik, um Ihre Triggergeräusche zu vermeiden?)**
0: Keine bewusste Vermeidung
1: Minimale Vermeidung. Weniger als 1 h am Tag oder nur gelegentlich
2: Mäßige Vermeidung. 1–3 h täglich oder häufige Vermeidung
3: Akute Vermeidung. Zwischen 3 und 8 h täglich oder sehr häufige Vermeidung
4: Extreme, extensive Vermeidung. Mehr als 8 h täglich. Konstante Vermeidung der Triggergeräusche
**Abschließend:** **Was wäre das schlimmste Szenario, wenn sie Ihre misophonischen Trigger nicht vermeiden könnten?**
*Schweregrad:*
0–4: subklinisch, d. h. kein Bedarf für ärztliche Behandlung
5–9: gering
10–14: mäßig
15–19: akut
20–24: extrem

Da ein psychiatrisches Syndrom Basis oder Kofaktor bei der Misophonie sein kann, ist eine entsprechende psychiatrisch-fachärztliche Begutachtung wichtig, auch zur Frage einer möglichen Therapieplanung (z. B. Verhaltenstherapie, medikamentöse Unterstützung bei Angststörung, Depression u. a.).

## Bewältigungsstrategien

Als Bewältigungsstrategien sind das Vermeiden von oder Sichentfernen aus Triggersituationen, das Nachahmen von Auslösegeräuschen zum „Aufheben“ oder „Gegensteuern“, das Verwenden von Ohrstöpseln, Kopfhörern oder Hören von Musik, Ablenken, Rezitieren, positiver interner Dialog zur Beruhigung, andere aufzufordern, keine Geräusche mehr zu machen, sowie gewissenhaft mit den eigenen Geräuschen umzugehen, bekannt [44, 55, 63].

Seebeck und Dozier 2019 führten Bewältigungsstrategien an, die zwar nicht misophone Reflexe ändern, aber deren Auswirkung reduzieren können: Offen über Misophonie reden, eine triggerfreie Oase schaffen (einschließlich Kopfhörer mit Geräuschunterdrückung sowie Rausch-App, um Trigger zu blockieren), auf Wohlbefinden achten wegen dann geringerer Beeinträchtigung durch Trigger, Bewältigungspläne für die ganze Familie erstellen [59].

## Therapie

Eine standardisierte Therapie der Misophonie ist bisher nicht bekannt. Die Therapie beginnt mit der Benennung der Misophonie, was für die Betroffenen ein erster Schritt in der Bewältigung darstellen kann. Allgemein fußt die Therapie multimodal auf Aufklärung von möglichen Zusammenhängen, Informationen zu dieser besonderen Geräuschüberempfindlichkeit und mögliche Bewältigungsstrategien/Alltagsmodifizierungen, auch zu möglichem Berufswechsel und über die psychiatrische/psychologische Expertise.

Eine medikamentöse Therapie gibt es aktuell nicht, jedoch könnte bei komorbider psychischer Störung deren medikamentöse Behandlung auch die Misophoniereaktion reduzieren. Angst kann signifikanter Mediator bzgl. der Beziehung zwischen Misophonie und Wutausbrüchen sein [67, 68]. Da selektive Serotonin-Wiederaufnahmehemmer (SSRI) auch Angst vermindern können, stellen sie möglicherweise eine Therapieoption bzgl. aggressiver Impulse bei Misophonie dar. Seebeck berichtete im Jahr 2016, dass sein Sohn mit 18 Jahren in eine Depression gerutscht sei, die mit Citalopram behandelt worden sei, das die Empfindlichkeit Geräuschen gegenüber etwas zu verbessern schien [57]. 2019 äußerte Seebecks Sohn „Seit ich Medikamente dagegen nehme, ist wenigstens die extreme Wut weg.“ [63].

Technisch-elektronische Hilfsmittel zur Dämpfung der Triggerlautstärke sind Kopfhörer, zur Maskierung (z. B. Musik hören über Kopfhörer oder im Raum, z. B. „Zimmerbrunnen“) sowie professionelle Tinnitusmasker mit individuell angepassten Otoplastiken im Sinne von „Noisern“ [53]. Tinnitusmasker („Noiser“) generieren entweder Umgebungs- oder individuell maßgeschneiderte Geräusche. Trotz der häufigen Verwendung dieser technischen Maskierungsverfahren liegen nur begrenzt Daten aus kontrollierten Studien vor [38].

## Neuromodulatorische Therapieansätze

Bisher publizierte oder von Einzelpersonen entwickelte Verfahren umfassen häufig in Kombination Verhaltenstherapien [4, 43, 48, 56, 1], die Gegenkonditionierung [15] und audiologisch-technische Verfahren. Bei der *kognitiven Verhaltenstherapie* (KVT, CBT, „cognitive behavioral therapy“) [4, 15, 26, 43, 48] ist das Ziel das Sich-bewusst-Machen maladaptiver Muster [38], Überprüfung von Schlussfolgerungen auf ihre Angemessenheit, Korrektur von irrationalen Einstellungen und Transfer korrigierter Einstellungen ins konkrete Verhalten. Basis für diese Behandlung ist die reduzierte kognitive Kontrolle bei Misophonikern während der Exposition zu Triggergeräuschen [13, 50]. Ist die Kognition inadäquat (z. B. durch Wahrnehmungsselektion und -bewertung), ist auch die Möglichkeit beeinträchtigt, Affekt und Verhalten zu korrigieren [16, 56].

Die von Jastreboff ursprünglich entwickelte *Tinnitus-Retraining-Therapie (TRT*) zur Tinnitusbehandlung [30, 32, 33], findet auch bei der Misophoniebehandlung Anwendung. Sie ist eine Kombination von Counseling (Information/Unterrichtung zur Hörpathologie) und auditorischer Stimulation durch Masker bzw. Hörgeräte. *Die Tinnitus-Bewältigungstherapie (TBT)* stellt eine Modifikation der Tinnitus-Retraining-Therapie dar. Sie stützt sich vorrangig auf kognitiv-behaviorale Interventionen: Edukation, Vermittlung von Aufmerksamkeitslenkungsstrategien, kognitive Umstrukturierung, Vermittlung allgemeiner Stress- und Tinnitusbewältigungsstrategien, Abbau von Vermeidungsverhalten und Entspannungsmethoden. Einige Studien belegen positive Effekte der Methoden [38]. Es fehlen qualitativ hochwertige kontrollierte Studien.

Seebeck und Dozier führen weitere Behandlungsmöglichkeiten auf: *Die Neural-Repatterning-Technique (NRT,* Gegenkonditionierung des misophonen Reflexes durch minimale Darbietung des Triggers, entscheidender Muskelreflex wird unterdrückt, misophone Rektion bleibt aus),* Sequent-Repatterning-Hypnotherapie *(SRT; Unterstützung emotionale Kontrolle trotz körperlicher Reaktion auf Trigger), *Trauma Buster Technique* (TBT, Reduktion Triggerreaktion durch gezielte Triggergeräuschverfremdung und Klopfakupressur), *Trauma and Tension Releasing Exercises (TRE*, Muskelreflexreduktion bei Trigger) [60].

Sämtliche dieser neuromodulatorischen Ansätze sind trotz vielversprechender Erfahrungsberichte derzeit als experimentell zu betrachten. Weitere Verfahren sollen die aktive Entspannung fördern wie z. B. die Progressive Muskelentspannung nach Jacobson, Yoga und Meditation, auch Hypnose kann wirksam sein.

Eine Übersicht über technische Hilfsmittel wie Rauschgeneratoren, Kopfhörer, Apps u. a.:

- https://misophonia-association.org,
- https://misophonie.de

Hilfreiche Adressen:

- https://misophoniainstitute.org
- https://misophoniatreatment.com
- https://misophonie.de
- www.misophoniehilfe.de

## Fazit für die Praxis

- Kinder, ihre Eltern und Erwachsene mit Misophonie wissen oftmals nicht, wie ihr Geräuschempfinden benannt werden kann: denn wer erklärt, er platze vor Wut, wenn die Partner/die Eltern beim Essen laut kauen/schlucken, erhält eher selten eine Diagnose, sondern eher ein ungläubiges Staunen oder Unverständnis.
- Die HNO-ärztliche Vorstellung von Misophoniebetroffenen ist primär die Beurteilung einer Hörstörung, auch im Hinblick auf eine mögliche Hyperakusis/Phonophobie oder einen Tinnitus, in Einzelfällen auch der Ausschluss einer auditiven Verarbeitungs-/Wahrnehmungsstörung.
- Die Ursachen sind letztendlich bis heute ungeklärt, die Diagnostik nicht standardisiert und eine (evidenzbasierte) Therapie zu diesem Zeitpunkt nicht bekannt. Wichtiges Standbein ist aber die Initiierung auch einer psychiatrischen Beurteilung/Unterstützung sowie eine HNO-Beratung über mögliche Behandlungskonzepte. Eine Verbesserung mit „Auswachsen“ der Misophonie ist bei praktiziertem Nihilismus nicht zu erwarten.
